# Comprehensive analysis of key genes and microRNAs in radioresistant nasopharyngeal carcinoma

**DOI:** 10.1186/s12920-019-0507-6

**Published:** 2019-05-28

**Authors:** Ya Guo, Yang Zhang, Shu Juan Zhang, Yi Nan Ma, Yun He

**Affiliations:** 1 Department of Oncology, The Second Affiliated Hospital of Medical College, Xi’an Jiao Tong University, 157 xi wu road, Xi’an, 710004 People’s Republic of China; 2 Department of Oncology, Kashi No.2 peoples’ Hospital of Xin Jiang, Kashi, 844000 Xin jiang China

**Keywords:** Nasopharyngeal carcinoma, microRNA, gene expression omnibus differentially expressed genes, bioinformatics analysis

## Abstract

**Background:**

Radioresistance is one of the main obstacle limiting the therapeutic efficacy and prognosis of patients, the molecular mechanisms of radioresistance is still unclear. The purpose of this study was to identify the key genes and miRNAs and to explore their potential molecular mechanisms in radioresistant nasopharyngeal carcinoma.

**Methods:**

In this study, we analysis the differentially expressed genes and microRNA based on the database of GSE48501 and GSE48502, and then employed bioinformatics to analyze the pathways and GO terms in which DEGs and DEMS target genes are involved. Moreover, Construction of protein-protein interaction network and identification of hub genes. Finally, analyzed the biological networks for validated target gene of hub miRNAs.

**Results:**

A total of 373 differentially expressed genes (DEGs) and 14 differentially expressed microRNAs (DEMs) were screened out. The up-regulated gene JUN was overlap both in DEGs and publicly available studies, which was potentially targeted by three miRNAs, including hsa-miR-203, hsa-miR-24 and hsa-miR-31. Moreover, Pathway analysis showed that both up-regulated gene and DEMs target genes were enriched in TGF-beta signaling pathway, Hepatitis B, Pathways in cancer and p53 signaling pathway. Finally, we further constructed protein-protein interaction network (PPI) of DEGs and analyzed the biological networks for above mentioned common miRNAs, the result indicated that JUN was a core hub gene in PPI network, hsa-miR-24 and its target gene were significantly enriched in P53 signaling pathway.

**Conclusions:**

These results might provide new clues to improve the radiosensitivity of Nasopharyngeal Carcinoma.

**Electronic supplementary material:**

The online version of this article (10.1186/s12920-019-0507-6) contains supplementary material, which is available to authorized users.

## Background

Radiotherapy is a mainly treatment for nasopharyngeal carcinoma (NPC). However, radioresistance is one of the major factors to affect the therapeutic efficacy and prognosis of patients [1–3]. Accordingly, identifying potential biomarkers and studying the molecular mechanisms associated with radioresistant nasopharyngeal carcinoma has become a hot topic both in basic and clinical research.

Microarrays are considered to be an important method for identifying potential biomarkers in many diseases at the molecular level with more effective and detailed insights [4]. Several microRNAs and mRNAs have been discovered to be involved in radioresistant NPC, whereas traditional methods have failed to elucidate the interaction of mRNAs and microRNAs and the molecular mechanisms of NPC due to the limitations on the comparative analysis [5–7]. Therefore, systematically investigating the interaction between microRNA and mRNA, and elucidating the molecular mechanism of radioresistant NPC is of great significance. With the development of bioinformatics, we can apply global analysis to process the data generated by microarray technology and find the interaction between DEGs and DEM, especially in the pathway interaction network, to summarize their potential mechanisms in diseases [8–10]. Based on above mentioned reasons, the present study aims to identify the key genes and miRNAs and to explore their potential molecular mechanisms in radioresistant nasopharyngeal carcinoma.

In this study, we analysis the differentially expressed genes and microRNA between radioresistant NPC CNE2-R cells and radiosensitive CNE2 cells based on the database of GSE48501 and GSE48502, and then employed bioinformatics to analyze the pathways and GO terms in which DEGs and DEMS target genes are involved. Moreover, Construction of protein-protein interaction network and identification of hub genes. Finally, analyzed the biological networks for validated target gene of hub miRNAs. Our data may provide an important contribution to identified biological markers and clarify the mechanisms of NPC radioresistance.

## Results

### DEGs and DEMs in radioresistant NPC cells compared with radioresistant NPC cells

**Fig. 1:**
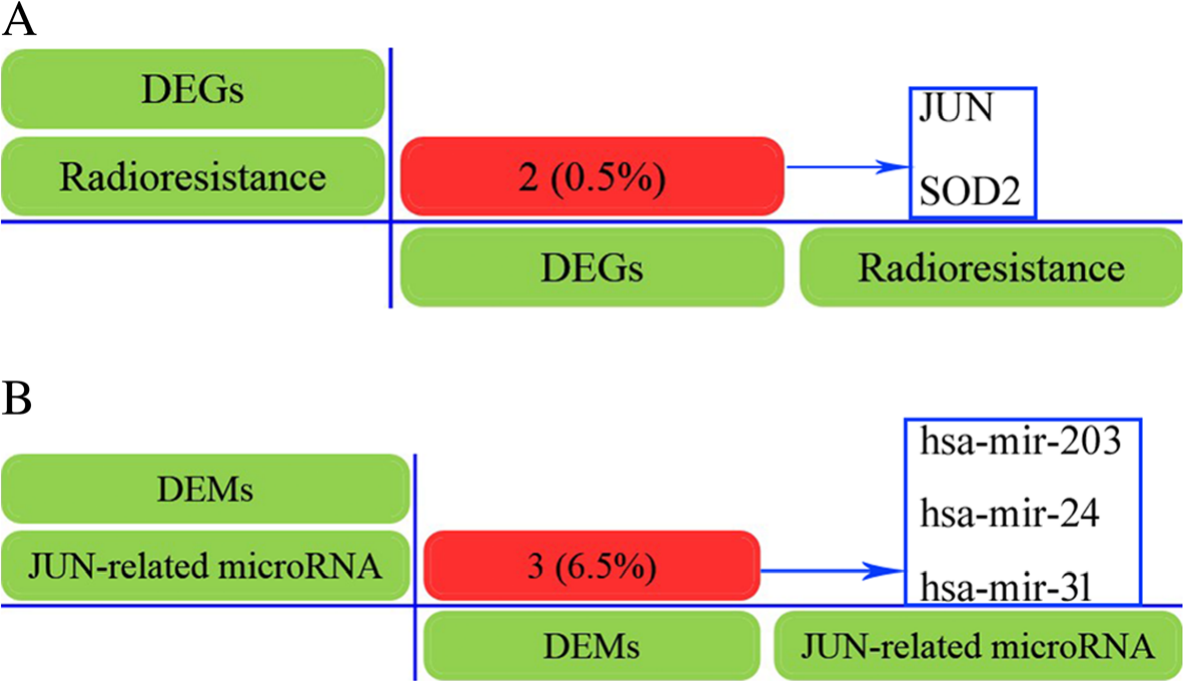
Screening common genes or miRNAs by Venn diagram software. **a** Identification common genes between the DEGs and the publicly available studies by Venn diagram. **b** Analyzed the common microRNAs between the JUN-related microRNAs and DEMs by Venn diagram software

GEO2R analyzed result shown that a total of 373 DEGs were identified in radioresistant NPC cells, including 291 mRNAs were up-regulated and 82 mRNAs were down-regulated (Table 1). The DEMs results indicated that there were 277 miRNAs were detected, 14 of which were differentially expressed with≥1.5 fold-change (t-test, *P* < 0.05), including 4 up-regulated miRNAs and 10 down-regulated miRNAs (Table 2). Moreover, DigSee software were used to identify the radioresistant related genes for publicly available studies, 37 related genes were retrieved. In addition, Venn diagram analyses revealed that JUN and SOD2 were common both in the DEGs and the DigSee (Fig. 1a). Furthermore, we identified JUN related microRNA by mirDIP software and analyzed the common microRNAs between the JUN-related microRNAs and DEMs by Venn diagram software. 35 JUN-related microRNA were retrieved, 3 down-regulated microRNAs were detected which were joint in JUN-related microRNAs and DEMs, including hsa-miR-203, hsa-miR-24 and hsa-miR-31 (Table 3 and Fig. 1b).

**Table 1:** Differential mRNA expression profile of radioresistant nasopharyngeal carcinoma CNE2R versus CNE-2 cells (The Table 1 show the top 20 differential expression genes)

Gene Symbol	Description	Fold Change
LXN	latexin	22.53
IGFBP3	insulin-like growth factor binding protein 3	18.88
ABCG1	ATP-binding cassette, sub-family G (WHITE), member 1	16.82
CP	ceruloplasmin (ferroxidase)	14.76
TRIM31	tripartite motif-containing 31	12.30
NNMT	nicotinamide N-methyltransferase	10.96
GDF15	growth differentiation factor 15	10.15
INHBE	inhibin, beta E	9.59
EGR1	early growth response 1	7.95
IL8	interleukin 8	7.49
METTL7A	methyltransferase like 7A	7.31
LOC387763	hypothetical LOC387763	7.24
LCN2	lipocalin 2	6.82
EDN2	endothelin 2	6.57
BMP2	bone morphogenetic protein 2	6.56
C8orf4	chromosome 8 open reading frame 4	6.42
ASNS	asparagine synthetase	6.12
SLC16A6	solute carrier family 16, member 6 (monocarboxylic acid transporter 7)	5.55
PCK2	phosphoenolpyruvate carboxykinase 2 (mitochondrial)	5.44
STEAP4	STEAP family member 4	5.32

**Table 2:** Differentially expressed miRNAs in GSE48502

miRNA	Fold change	P-value
Up-regulated miRNA		
hsa-miR-762	2.510	0.00337
hsa-miR-1202	2.292	0.0008
hsa-miR-193b	1.530	0.00986
hsa-let-7e	1.521	0.00054
Down-regulated miRNA		
hsa-miR-203	3.337	0.01698
hsa-miR-545	1.980	0.04888
hsa-miR-4291	1.722	0.00271
hsa-miR-183	1.677	0.03486
hsa-miR-24	1.667	0.00032
hsa-miR-130a	1.598	0.01252
hsa-miR-660	1.578	0.01531
hsa-miR-31	1.535	0.00208
hsa-miR-23a	1.527	0.03552
hsa-miR-30a	1.526	0.0274

**Table 3:** Identification of JUN related microRNA by mirDIP software. Prediction analysis was performed by mirDIP online software. In this table, asterisk represents common microRNA in DEMs and JUN-related microRNAs by Veen analysis

Gene Symbol	MicroRNA	Integrated Score	Score Class
JUN	hsa-miR-200b-3p	0.8428	Excellent
JUN	hsa-miR-139-5p	0.7769	Excellent
JUN	hsa-miR-200c-3p	0.7693	Excellent
JUN	hsa-miR-429	0.7576	Excellent
JUN	hsa-miR-495-3p	0.7162	Excellent
JUN	hsa-miR-32-5p	0.6837	Excellent
JUN	hsa-miR-92a-3p	0.6745	Excellent
JUN	hsa-miR-216b-5p	0.6528	Excellent
JUN	hsa-miR-522-3p	0.6392	Excellent
JUN	hsa-miR-501-5p	0.60082	Excellent
JUN	hsa-miR-200a-3p	0.5751	Excellent
JUN	hsa-miR-524–5p	0.5637	Excellent
JUN	hsa-miR-520d-5p	0.5365	Excellent
JUN	hsa-miR-141–3p	0.5211	Excellent
JUN	hsa-miR-203*	0.5019	Excellent
JUN	hsa-miR-580-3p	0.4817	Excellent
JUN	hsa-miR-940	0.4770	Excellent
JUN	hsa-miR-1299	0.4628	Excellent
JUN	hsa-miR-9-5p	0.4390	Excellent
JUN	hsa-miR-612	0.4313	Excellent
JUN	hsa-miR-583	0.4260	Excellent
JUN	hsa-miR-455-3p	0.4018	Excellent
JUN	hsa-miR-637	0.3870	Excellent
JUN	hsa-miR-92b-3p	0.3700	Excellent
JUN	hsa-miR-758-3p	0.3659	Excellent
JUN	hsa-miR-25-3p	0.3602	Excellent
JUN	hsa-miR-24*	0.3585	Excellent
JUN	hsa-miR-31*	0.3585	Excellent
JUN	hsa-miR-493-5p	0.3318	Excellent
JUN	hsa-miR-127-5p	0.3255	Excellent
JUN	hsa-miR-633	0.3227	Excellent
JUN	hsa-miR-766-3p	0.3199	Excellent
JUN	hsa-miR-224-3p	0.3097	Excellent
JUN	hsa-miR-494-3p	0.3081	Excellent
JUN	hsa-miR-1285-3p	0.3039	Excellent

### Gene ontology analysis of DEMs target genes and DEGs

**Fig. 2:**
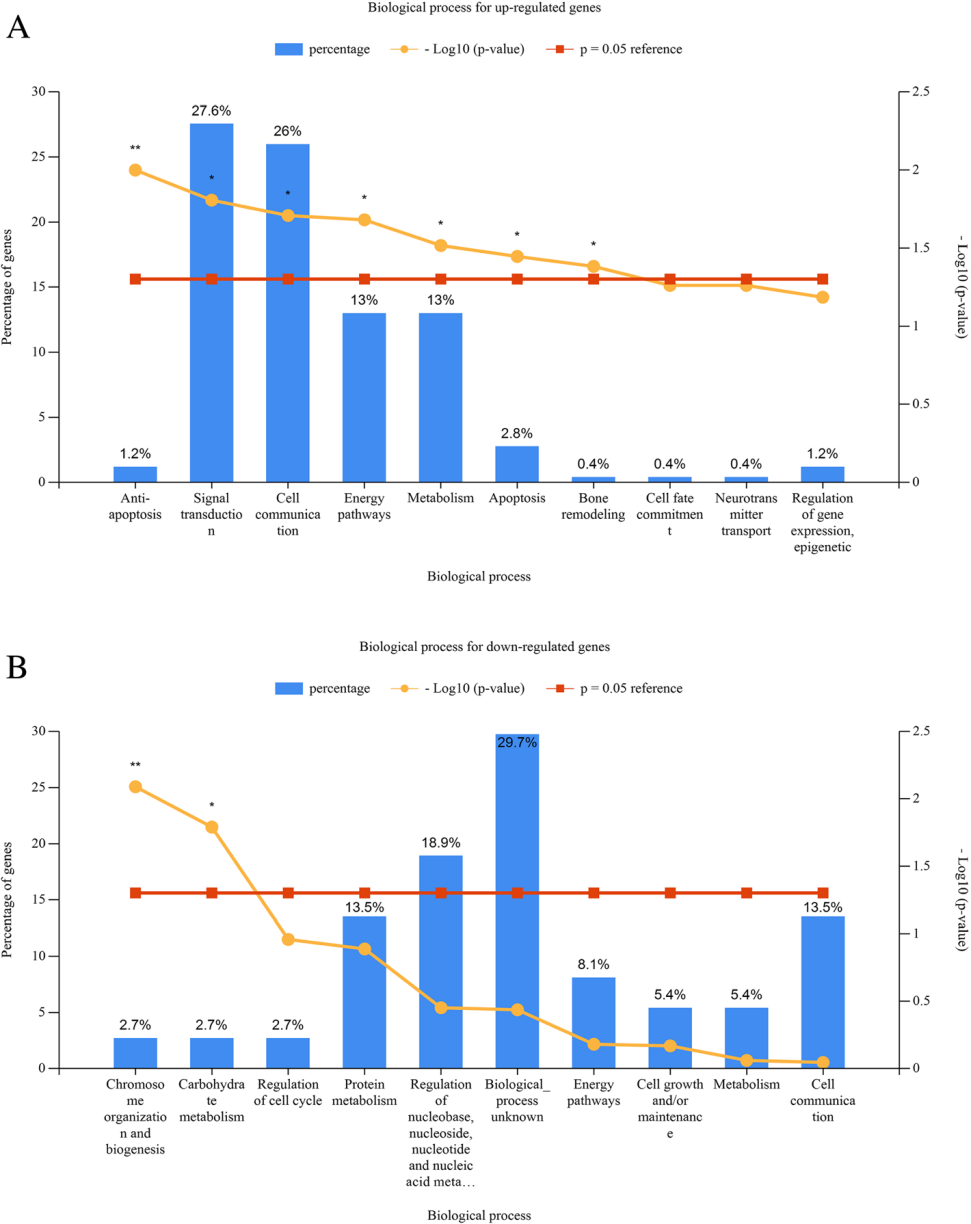
GO functional annotation of DEGs. **a** The top 20 significant biology process of up-regulated genes. **b** The significant biology process of down-regulated genes

We performed gene ontology (GO) analysis of DEGs and DEMs target genes. Our result indicated that the significantly enriched GO terms of up-regulated and down-regulated microRNAs target genes were mainly involved in mitotic cell cycle; RNA binding; nucleoplasm; cytosol; biosynthetic process; gene expression; cellular nitrogen compound metabolic process; ion binding (Table 4). As shown in Fig. 2, the most significantly enriched GO terms corresponded to up-regulated DEGs were “Anti-apoptosis” (Ontology Biological Process), the most significant biological process for the down-regulated genes are Chromosome organization and biogenesis.

**Table 4:** GO functional annotation of DEMs (Top 10)

GO Category	Gene Target	miRNAs	P-value
Up-regulated miRNAs			
mitotic cell cycle	158	4	0
protein binding transcription factor activity	143	4	0
RNA binding	473	4	0
nucleoplasm	369	4	0
cytosol	664	4	0
biosynthetic process	909	4	0
gene expression	234	4	0
viral process	177	4	0
cellular nitrogen compound metabolic process	1112	4	0
ion binding	1145	4	0
Down-regulated miRNAs			
nucleoplasm	612	18	0
biosynthetic process	1636	18	0
gene expression	389	18	0
cellular nitrogen compound metabolic process	2032	18	0
organelle	3947	18	0
ion binding	2198	16	0
mitotic cell cycle	221	15	0
RNA binding	812	15	0
cellular protein modification process	985	15	0
cytosol	1170	15	0

### Pathway enrichment analyses

**Fig. 3:**
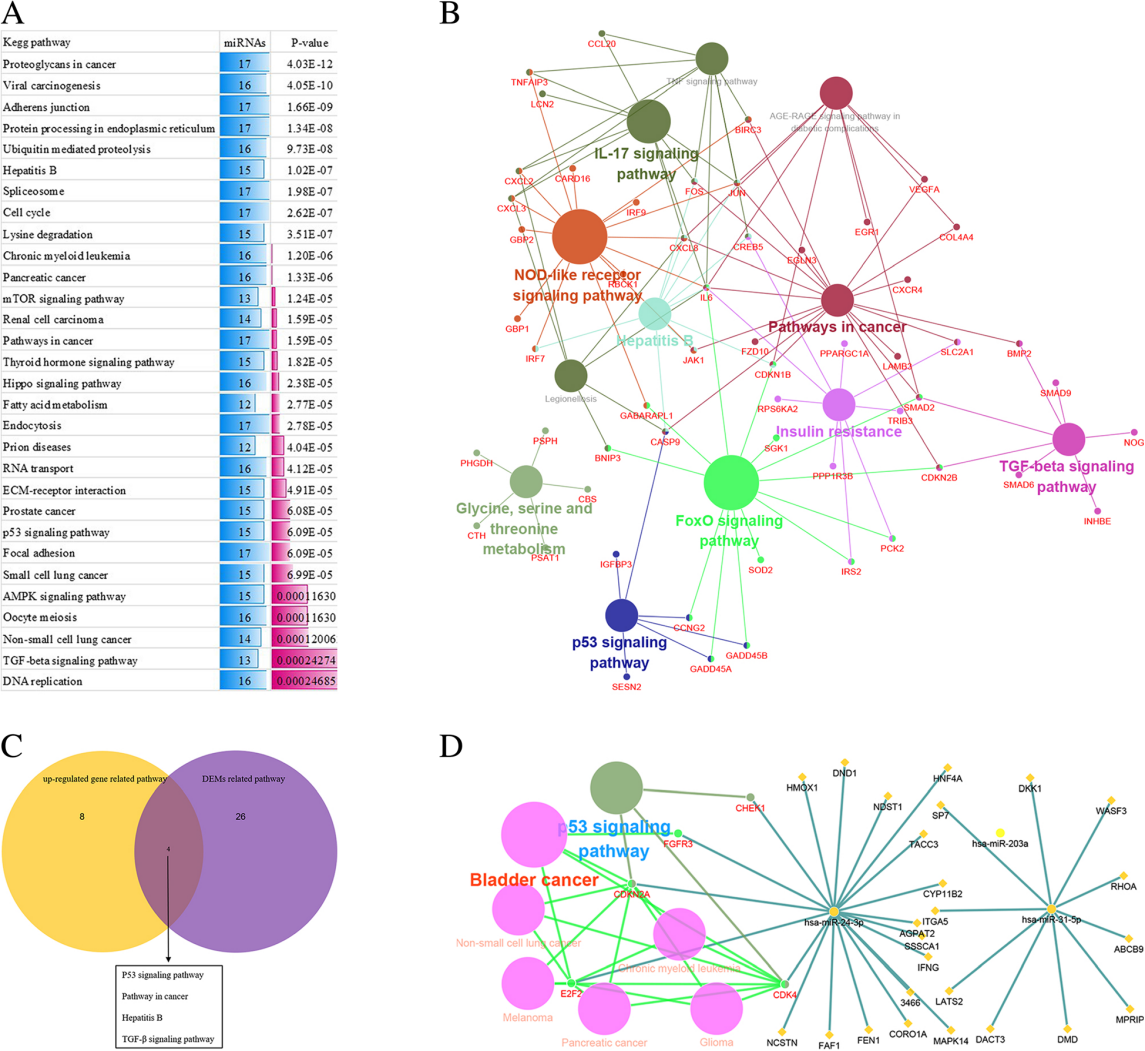
Pathway enrichment analysis of DEMs target genes and DEGs. **a** The top 30 enriched kegg pathway for DEMs target genes. **b** Significant pathways in up-regulated genes. **c** Venn diagrams show the common pathway between upregulated genes and DEMs target genes. **d** Biomolecular network about 5 validated genes (in red) targeted by the common microRNAs and corresponding pathways were analyzed by Clue Go and Clue Pedia. The yellow diamond nodes represent target gene, the violet circle and red circle nodes represent miRNA and their related pathway respectively

We performed pathways enrichment analysis of DEMs target genes and DEGs using DIANA miRPATH tool and Clue go software respectively. For the pathway analysis, the top of 30 significant pathways were selected in the DEMs target genes (Fig. 3a). Moreover, the upregulated DEGs were enriched in 12 kegg pathways (Fig. 3b). We further identified 4 significant pathways both in up-regulated DEGs and DEMs, including TGF-beta signaling pathway, Hepatitis B, Pathways in cancer and p53 signaling pathway which were considered as crucial pathways (Fig. 3c). Finally, we analyzed the above mentioned microRNAs (hsa-miR-203, hsa-miR-24 and hsa-miR-31) and their corresponding pathways using Clue go and Clue pedia software. The results indicated that hsa-miR-24 and 20 target genes were associated with 7 pathways, and the P53 signaling pathway is the most significant pathway (Fig. 3d). Our result indicated that P53 signaling pathway may be related to nasopharyngeal carcinoma radioresistance.

### Protein-protein interaction network and subnetwork of DEGs

**Fig. 4:**
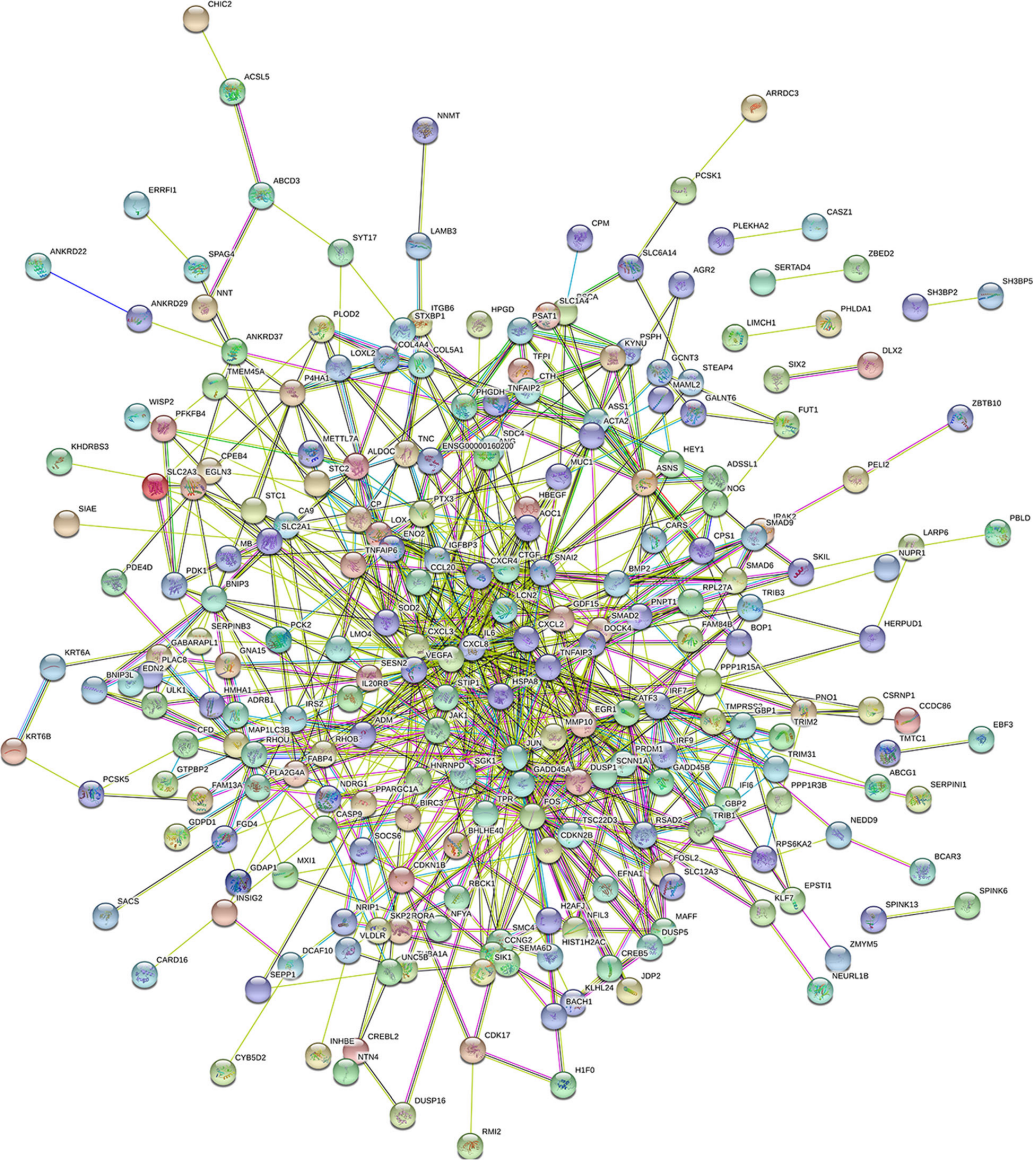
Constructed PPI network of DEGs by STRING software. Using the STRING software, proteins are represented with nodes and the interactions with continuous lines to represent direct interactions (physical), while indirect ones (functional) are presented by interrupted lines. Line thickness indicates the strength of data support

**Fig. 5:**
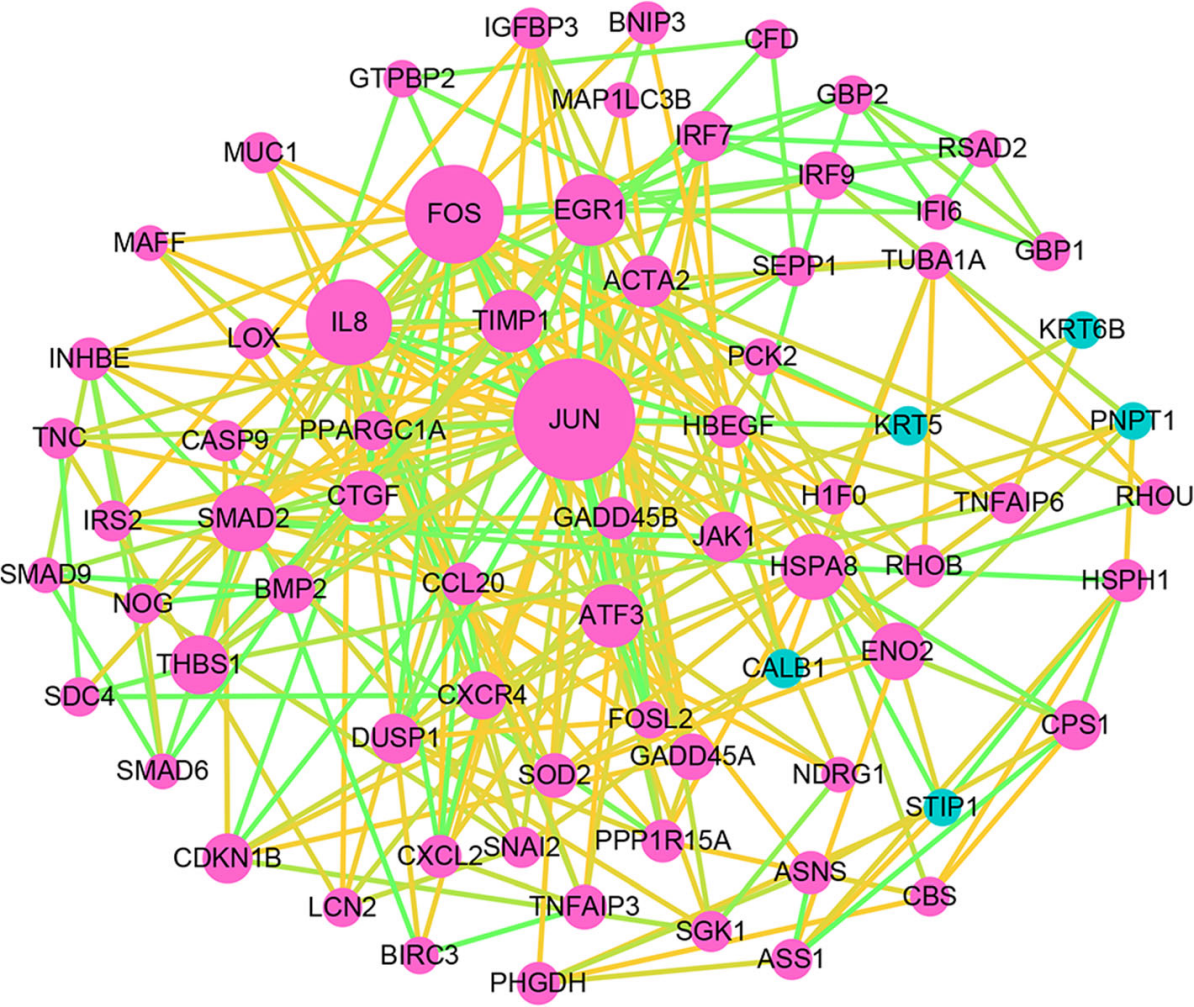
Significant subnetwork of DEGs. Red nodes represent up-regulated genes, while Green nodes denote down-regulated genes. The size of the nodes is positively correlated with the count of genes. The color of line is determined by the combined score provided by STRING

The PPI network was consisted of 339 nodes and 714 edges which were mapped by STRING software (Fig. 4). As shown in Fig. 5, the PPI network of DEGs was composed of 72 nodes that were interacted with each other. The connectivity degree of each node was calculated in this PPI network and the top 5 nodes with degree more than 20 were JUN, FOS, IL8, EGR, HSPA8. Among these genes, JUN with highest degree (54) in the Protein-Protein interaction network was considered as the hub node which have closely interacted with other genes. Therefore, we can infer that the up-regulated JUN may be a key node related with radioresistant nasopharyngeal carcinoma.

## Discussion

Nasopharyngeal carcinoma is a geographically distributed disease, especially in southern china and southeast Asia. Although radiotherapy is considered to be the primary treatment for NPC, radioresistance-induced locoregional recurrence and distant metastases remains mainly obstacle to successful treatment [11, 12]. Therefore, in-depth study biomarkers and mechanisms of radioresistance in NPC is of great significance for improving the radiosensitivity of NPC and provide a new ideas for design of good therapy.

Previous studies have identified that radioresistance-associated molecules (mRNAs, microRNAs, and proteins) regulate radioresistance through different biological process, such as DNA repair, apoptosis, cell cycle, and protective autophagy [9]. However, the molecular mechanisms underlying NPC radioresistnce remain elusive.

In order to understand the mechanisms underlying in radioresistant NPC, we performed global analysis of key genes and microRNAs in radioresistant Nasopharyngeal Carcinoma by bioinformatics analysis. Our result demonstrated that a total of 373 DEGs and 14 DEMs between radioresistant NPC CNE2-IR cells and radiosensitive CNE2 cells were identified. Our study revealed that JUN was significantly up-regulated in radioresistant NPC CNE2-IR cell and which was overlap between the DEGs and the DigSee. Meanwhile, in Protein-Protein interaction network analysis, JUN was a core hub gene in PPI network. Recent research has shown that JUN is an important components of the activator protein-1 (AP-1) transcription factor and is closely related to cell proliferation, apoptosis and malignant transformation [13]. JUN could promote tumor growth and progression. Over-expression of c-jun was found to result in abnormal cell proliferation and loss of apoptosis. Some researchers have noted that inhibition the expression of c-jun can enhances radiosensitivity, induces cell cycle arrest and apoptosis [14]. Our previous study shown that the expression of c-jun was significantly up-regulated in CNE-2R cells, which may be associated with the radioresistance of NPC [15]. The kegg pathway analysis data indicated that JUN is involved in a variety of pathways. Such as TNF signaling pathway, Epstein-Barr virus infection, MAPK signaling pathway, Pathways in cancer. MAPK signaling activity confers inherent radioresistance to KRAS-mutant colorectal carcinoma cells by rapidly upregulating of heterogeneous nuclear ribonucleoprotein K (hnRNPK) [16]. Our result showed that JUN was involved in Hepatitis B, NOD-like receptor signaling pathway, AGE-RAGE signaling pathway in diabetic complications, Pathways in cancer, IL-17 signaling pathway, TNF signaling pathway (Fig. 3b). We speculated that JUN may be participated in the regulation of nasopharyngeal carcinoma radiosensitivity through these pathways, the internal mechanism may need to be further tested in the future experiments. Based on the above, we conclude that JUN is involved in NPC radioresistance, which may provide new clues to improve radiosensitivity of Nasopharyngeal Carcinoma.

microRNAs are single stranded, endogenous, 19–25 nucleotide (nt), which are thought to be modulated tumor radiosensitivity [17]. Therefore, identification of radioresistance associated miRNAs which may contribute to more effective treatments for NPC patients. In this study, we screened out 14 differentially expressed miRNAs in the radioresistant CNE2-R cells, including the up-regulated mirRNA-762, mirRNA-1202, mirRNA-193b, mirRNA-let-7e and down-regulated mirRNA-203, mirRNA-545, mirRNA-4291, mirRNA-183, mirRNA-24, mirRNA-130a, miRNA-660, miRNA-31, miRNA-30a, miRNA-23a, suggesting that the regulation of these miRNAs might be participate in the NPC radioresistance. Most of them have been shown to be associated with radioresistance of tumor [2, 18, 19]. mirRNA-let-7e and miRNA-31 have been recently discovered to involving in the acquisition of cancer cell radioresistance [1, 19]. Recent reports indicate that miRNA-23a is downregulated in the radioresistant NPC tissues, and is an independent predictor of poor prognosis in patients with nasopharyngeal carcinoma. up-regulated miRNA-23a improves NPC cell radiosensitivity in vivo and vitro. Downregulated miRNA-23a increases NPC radioresistance through activating IL-8/Stat3 signaling. Targeting miR-23a/IL-8/Stat3 signaling might be an effective approach to improving radiosensitivity of NPC [18]. It has been reported that miR-24 is frequently downregulated in NPC cell lines, and the consumption of miR-24 inhibited NPC cell growth and proliferation, while improving the radiosensitivity of NPC both in vitro and in vivo. In addition, it is reported that SP1 was verified as a target for miR-24, miR-24/SP pathway should help us understand the radiosensitivity mechanisms of human NPC, which may be a potential therapeutic target [20]. Our study detected that miRNA-24 is down-regulated in radioresistant NPC cells, and which not only was common in DEMs and JUN-related microRNAs, but also had significantly enriched in P53 signaling pathway. In conclusion, we can infer that the above mention microRNAs, especially miRNA-24 may be a key factor to affect the radiosensitivity of NPC, which may be helpful to predict radiosensitivity in NPC.

To completely understand the function of miRNAs and mRNAs in radioresistant NPC, we performed pathway enrichment analysis of DEGs and DEMs, the result demonstrated that 4 pathways are considered as the key pathways for the radiosensitivity of NPC, including TGF-beta signaling pathway, Hepatitis B, Pathways in cancer and p53 signaling pathway, which were regulated by miRNA and mRNA together. Moreover, hsa-miR-24 and its target gene were found have significantly enriched in P53 signaling pathway. P53 can predict cancer response to IR and chemotherapy [21]. Improving the radiosensitivity of non-small cell lung cancer cells by inhibition of TGF-β1 signaling [22]. It is reported that p53 signaling pathway correlates with the radioresponse of non-small cell lung cancer. Differentially expressed genes in the p53 signaling pathway related to DNA damage repair, apoptosis, cycle regulation, metastasis, deterioration and radioresistance [23]. Previous study has shown that p53 signaling pathway mediate inhibition and apoptosis induced by 12C6+ heavy ion beam irradiation on HepG2 cancer cells [24]. Accordingly, we can infer that hsa-miR-24 and p53 signaling pathway should provide an important contribution to understand the mechanisms of radiosensitivity in human NPC and that it may represent a potential therapeutic target.

## Conclusion

In conclusion, this study demonstrates that the upregulated gene JUN was overlap both in DEGs and publicly available studies, was a core hub gene in PPI network, which was potentially targeted by three miRNAs, including hsa-miR-203, hsa-miR-24 and hsa-miR-31. Pathway analysis showed that both up-regulated gene and DEMs were enriched in TGF-beta signaling pathway, Hepatitis B, Pathways in cancer and p53 signaling pathway. Finally, we analyzed the biological networks for validated target gene of common miRNAs, the result indicated that miR-24 is frequently down-regulated in radioresistant NPC cell lines and significantly enriched in P53 signaling pathway. Based on these reasons, our study indicated that the JUN, miR-24 and P53 signaling pathway may be associated with radioresistance in Nasopharyngeal Carcinoma, and which may provide new clues for improving radiosensitivity in Nasopharyngeal Carcinoma. However, these results are only speculated by the combination of databases and bioinformatics methods, and still needs to be confirmed. In the following study, we explored the association between JUN expression and radioresistance in vitro. To further determine the clinical predictive value of JUN in NPC, we performed immunohistochemistry assays to examine the protein expression pattern of JUN in NPC specimens and normal nasopharyngeal epithelium specimens. And then, we performed a log-rank test analysis the Overall survival (OS) of patients with NPC based on JUN expression. Finally, Study the correlation between JUN expression and distant metastasis in patients with NPC.

## Methods

### Microarray data

The mRNA expression profile of GSE48501 and microRNA profile of GSE48502 were downloaded from the GEO database (Gene expression omnibus, http://www.ncbi.nlm.nih.gov/geo/) [7] (Additional file 1). As previousy described, the raw data was preprocessed by the application of bioconductor package ‘affy’ [6]. The sample of GSE48501 included 2 radioresistant NPC samples and 2 radiosensitive NPC in total, whereas the GSE48502 miRNA expression profiles included 3 radioresistant NPC samples and 3 radiosensitive NPC samples in total.

### Identification of DEGs and DEMs

GEO2R (http://www.ncbi.nlm.nih.gov/geo/geo2r/) is a web tool that can analyze almost GEO series. DEGs and DEMs were screened with GEO2R [25] (Additional file 1). The differentially expressed mRNAs were selected using adjusted P -values< 0.05 and |logFC| ≥ 1. *P* < 0.05 and fold-change≥1.5 were set as the cut-off criterion in the DEMs. Furthermore, we applied to the online tool Morpheus (https://software.broadinstitute.org/morpheus/) to generate a heat map of DEGs [26] (Additional file 1).

### Screening common genes between the DEGs and the publicly available studies by Venn diagram

We applied to disease gene search engine with evidence sentence (http://210.107.182.61/geneSearch/) to identify the radioresistant related genes for publicly available studies using the following keywords: “radioresistance” [9]. Then, the overlapping genes between the DEGs and the DigSee were screened by Venn diagram (http://bioinformatics.psb.ugent.be/webtools/Venn/) [27] (Additional file 1).

### Gene ontology and pathway analysis of DEGs and DEMs target genes

Gene ontology (GO) biological process terms and pathway enrichment analysis of differentially expressed genes was performed using FunRich software (www.funrich.org/) and Clue Go software respectively [10, 28] (Additional file 1). The *P*-value < 0.05 was considered significant GO and pathway term. DIANA miRPATH tool (http://www.funrich.org/) was used to analyze gene ontology and pathway analysis of DEMs target genes [29] (Additional file 1). *P*-value< 0.05 was set as the cut-off criterion in the significant GO terms and kegg pathway.

### PPI network construction and subnetwork mining

The STRING database (http://string-db.org/) is an online tool to construct protein-protein interaction network [30] (Additional file 1). The Cytoscape software is a tool for the visual exploration of interaction networks composed of protein, gene, and other types of interactions [31] (Additional file 1). In present study, the protein-protein interaction network (PPI) of DEGs was mapped by STRING and then visualized using Cytoscape. Combined score ≥ 0.4 was set as the cut-off criterion. The proteins with high degrees were considered as the hub nodes. In addition, we further constructed subnetwork mining in the PPI network based on CentiScape with centrality value is high/equals threshold 5.

### Hub genes related microRNAs were predicted using mirDIP online software

mirDIP online software (http://ophid.utoronto.ca/mirDIP/index.jsp) integrates twelve microRNA prediction datasets from six different microRNA prediction databases [32] (Additional file 1). In present study, hub genes corresponding microRNAs were predicted by mirDIP software. Top 5% was named high, Score class = high was set as the cut-off criterion.

### Analysis of biological networks for common miRNAs

Firstly, we used venn diagram to screen common microRNAs between JUN related microRNAs and DEMs. Then we established a regulatory network for common miRNAs, their target genes and pathways by Clue Go and Clue Pedia [1]. *P*-value≤0.05, cluster ≥3 and min genes ≥4% was named significant biological networks (Additional file 1).

## Additional file

Additional file 1: Related data in this article. (PDF 77 kb)

## Abbreviations

DEGs: Differentially expressed genes
DEMs: Differentially expressed microRNAs
GEO: Gene expression omnibus
GO: gene ontology
NPC: nasopharyngeal carcinoma
PPI: Protein-protein interaction network
