# Supplementary material for: Comprehensive analysis of key genes and microRNAs in radioresistant nasopharyngeal carcinoma
Source: BMC Med Genomics. 2019 May 28;12:73. doi: 10.1186/s12920-019-0507-6 (PMC6537399; doi:10.1186/s12920-019-0507-6)
Supplement: Supplementary file 1 — Related data in this article. (PDF 77 kb) [file 12920_2019_507_MOESM1_ESM.pdf]

1. The mRNA expression profile of GSE48501 and microRNA profile of GSE48502 were downloaded from the GEO database (Gene expression omnibus, http://www.ncbi.nlm.nih.gov/geo/).
2. GEO2R analysis software can be download from (http://www.ncbi.nlm.nih.gov/geo/geo2r/).
3. Disease gene search engine with evidence sentence (DigSee) can be got from (http://210.107.182.61/geneSearch/).
4. Venn diagram (http://bioinformatics.psb.ugent.be/webtools/Venn/).
5. Gene ontology (GO) biological process terms and pathway enrichment analysis of differentially expressed genes was performed using FunRich software (www.funrich.org/) and Clue Go software respectively. The P-value <0.05 was considered significant GO and pathway term. DIANA miRPATH tool (http://diana.imis.athena-innovation.gr/DianaTools/) .
6. The STRING database (http://string-db.org/) is an online tool to construct protein-protein interaction network, including physical and functional associations.
7. mirDIP online software (http://ophid.utoronto.ca/mirDIP/index.jsp).
8. Cytoscape software can be download from https://cytoscape.org/.
